# Supplementary material for: Cardiovascular Health Benefits of Exercise Training in Persons Living with Type 1 Diabetes: A Systematic Review and Meta-Analysis
Source: J Clin Med. 2019 Feb 17;8(2):253. doi: 10.3390/jcm8020253 (PMC6406966; doi:10.3390/jcm8020253)
Supplement: Supplementary file 1 [file jcm-08-00253-s001.pdf]

Literature search strategy: a MEDLINE example

1. exp Diabetes Mellitus, Type 1
2. exp Diabetic Ketoacidosis
3. exp Diabetes Complications
4. (IDDM or T1DM or T1D).ot,tw.
5. ((insulin depend or insulindepend) not (non-insulin depend or noninsulindepend)).ot,tw.
6. ((typ 1 or typ I or typ1 or typI) adj2 diabet).ot,tw.
7. ((acidos or juvenil or child or keto or labil or britt) adj2 diabet).ot,tw.
8. ((auto-immun or autoimmun or sudden onset) adj2 diabet).ot,tw.
9. (insulin defic adj2 absolut).ot,tw.
10. 1 or 2 or 3 or 4 or 5 or 6 or 7 or 8 or 9
11. exp Diabetes Insipidus
12. diabet insipidus.ot,tw.
13. 11 or 12
14. 10 not 13
15. exp Exercise
16. exp Exercise Therapy
17. exp Running
18. exp Swimming
19. exp Jogging
20. exp Sports
21. exp Physical activity
22. ((Weight or strength or resistance or circuit or aerob$) adj (lift$ or train$)).ab,ti.
23. ((strength$ or aerobic$ or anaerobic$) adj (exercise$ or train$)).ab,ti.
24. (physical adj activit$).ab,ti.
25. sport$.ab,ti.
26. exercis$.ab,ti.
27. exp Physical Fitness
28. (physical adj fitness).ab,ti.

29. (bicycling or cycling$ or swim$ or gym$ or walk$ or danc$ or yoga or tai chi or jogging or rambling or rowing or skate or skating or soccer or football or training or bicycling or calisthenic or cyclic or dance or dancing).ab,ti.

30. 15 or 16 or 17 or 18 or 19 or 20 or 21 or 22 or 23 or 24 or 25 or 26 or 27 or 28 or 29

31. exp clinical trial

32. exp randomized controlled trials

33. exp double-blind method

34. exp single-blind method

35. exp cross-over studies

36. randomized controlled trial.pt.

37. clinical trial.pt.

38. controlled clinical trial.pt.

39. (clinic$ adj2 trial).mp.

40. (random$ adj5 control$ adj5 trial$).mp.

41. (crossover or cross-over).mp.

42. ((singl$ or double$ or trebl$ or tripl$) adj (blind$ or mask$)).mp.

43. randomi$.mp.

44. (random$ adj5 (assign$ or allocat$ or assort$ or reciev$)).mp.

45. 31 or 32 or 33 or 34 or 35 or 36 or 37 or 38 or 39 or 40 or 41 or 42 or 43 or 44

46. 14 and 30 and 45
